# Editorial for the Special Issue ‘Molecular Breeding and Genetics Research in Plants’

**DOI:** 10.3390/cimb47010011

**Published:** 2024-12-29

**Authors:** Shimeles Tilahun

**Affiliations:** 1 Agriculture and Life Science Research Institute, Kangwon National University, Chuncheon 24341, Republic of Korea; shimeles@kangwon.ac.kr; 2 Department of Horticulture and Plant Sciences, Jimma University, Jimma 378, Ethiopia

Despite significant advancements in plant breeding research, the challenges posed by a growing global population, the impact of abiotic and biotic stresses, and the uncertainties of climate change necessitate continued focus and innovation in plant breeding and genetic studies. In recent decades, advancements in molecular breeding and genetics research in plants have significantly accelerated the crop breeding process. For instance, recent studies have advanced rice yield and quality by regulating grain size [1], enhanced crop growth through improved nitrogen use efficiency [2], and developed strategies to make crops more tolerant to stresses like cold, salinity, and drought, ensuring consistent yields [3,4,5]. Among the traits influencing rice yield, studies suggest a strong connection between grain filling and starch metabolism [6,7]. However, achieving high yields alongside optimal grain filling ratios in cultivated rice varieties remains challenging and requires continued research leveraging modern molecular biology technologies to address this issue effectively [8,9]. Lee et al. [10] reported several quantitative trait loci (QTLs) related to rice grain filling. In addition to the yields, as living standards and consumer expectations continue to rise, enhancing the quality of rice has become increasingly important [11]. Gong et al. [12] reviewed the significant progress made in identifying genes and QTL linked to essential traits such as appearance, aroma, texture, and nutritional value in rice. These genetic discoveries have enabled the development of molecular markers, which serve as powerful tools for marker-assisted selection, for the improvement of rice quality.

Similarly, advances in molecular breeding and genetics have significantly transformed important horticultural crops such as tomatoes. In tomatoes, the development of molecular biological techniques, such as QTL mapping techniques and genome-wide association studies (GWASs), has facilitated the understanding of the genetic architecture of complex traits and germplasm management of both wild and cultivated tomatoes [13]. Breeding efforts have focused on addressing key challenges such as productivity, fruit sensorial and nutritional quality, resistance to biotic and abiotic stresses, and adaptation to new growth conditions [13,14]. These insights into molecular mechanisms driving better yield, product quality, nutrient efficiency, and stress adaptation in rice and tomato underscore the importance of further innovations in other crops as well.

This Special Issue, ‘Molecular Breeding and Genetics Research in Plants’, in *Current Issues in Molecular Biology* showcases advancements in molecular breeding, genomics, and gene characterization aimed at improving crop yield, stress tolerance, and disease resistance, providing valuable insights for sustainable agriculture. In this Special Issue, 19 manuscripts, comprising 16 research articles, 2 reviews, and 1 brief report are included after rigorous evaluation by the editors and expert reviewers. Five key topics are addressed: (i) advances in molecular breeding and genetics; (ii) stress tolerance and environmental adaptation; (iii) plant health and resistance to weeds and diseases; (iv) anthocyanin biosynthesis and pigmentation; and (v) specialized functional studies.

Relating to the first topic (i), Tian et al. [15] developed a colorimetric loop-mediated isothermal amplification (LAMP) assay for rice to detect the dense and erect panicle 1 (dep1) allele, a gene critical for improving traits like yield, quality, and stress tolerance. Similarly, Tang et al. [16] identified and characterized 16 ZmFAR1 genes in maize (inbred line B73), highlighting their roles in light signaling and stress responses. Notably, ZmFAR1-14 and ZmFAR1-9 were linked to kernel row number and kernel weight, respectively, under shading conditions, emphasizing their potential for maize breeding. Furthering the genetic analysis of crops, Yan et al. [17] studied soluble solids content (SSC) in melon, identifying five candidate genes involved in sugar metabolism and accumulation, thereby offering valuable insights for breeding sweeter varieties. Meanwhile, Barbosa et al. [18] used whole-genome resequencing to uncover genetic markers for abaca varietal improvement, enhancing the identification and genotyping of this important fiber crop.

Regarding topic (ii), salinity, low-temperature tolerance, and environmental adaptability pose major challenges in agriculture. Li et al. [19] developed salt-tolerant rice varieties by incorporating Xian (*Indica*) alleles into Geng (*Japonica*) rice, leveraging the genetic diversity of Xian for high-yielding and salt-tolerant Geng varieties. In maize, Yu et al. [20] conducted a GWAS to identify SNPs associated with low-temperature tolerance, uncovering a key SNP on chromosome 5 that explained significant phenotypic variation. Addressing adaptation to low latitudes, Song et al. [21] used CRISPR-Cas9 to edit the EHD1 gene in *Japonica* rice, delaying flowering and improving adaptability while retaining superior grain quality and higher yields. Chen et al. [22] explored stress-specific miRNA expression in maize under salinity and alkalinity, revealing distinct molecular pathways linked to ion transport and metabolism. Further studies on stress resistance include Zhang et al. [23], who identified and characterized a novel gene, ClNUM1, and its splice variants in *Chrysanthemum lavandulifolium*, demonstrating varying levels of salt and cold tolerance. Guo et al. [24] identified the KvCHX gene in the halophyte *Kosteletzkya virginica*, which enhanced salinity tolerance and potassium homeostasis when overexpressed in Arabidopsis.

Concerning the third topic (iii), innovative strategies for combating biotic stressors are essential for sustainable agriculture. Gerakari et al. [25] studied broomrape resistance in tomatoes, identifying transcriptomic differences in introgression lines derived from *Solanum lycopersicum* and its wild relative *S. pennellii*, providing insights for molecular breeding and sustainable weed management in tomatoes and other crops. Hamidi et al. [26] focused on managing banded leaf and sheath blight in maize using salicylic acid (SA) and jasmonic acid (JA) as elicitors to boost resistance through induced host defense mechanisms. In viticulture, Kim et al. [27] developed an in vitro propagation method for ‘Shine Muscat’ grapevines, ensuring disease-free plants with confirmed genetic stability to support large-scale production.

Topic (iv) focuses on anthocyanin biosynthesis and pigmentation, as anthocyanins are fundamental to plant coloration. Fu et al. [28] identified IbERF1 as a key upstream regulator of the IbMYB1-4 promoter, which controls anthocyanin biosynthesis in purple-fleshed sweet potatoes. Wang et al. [29] reviewed black pigmentation in plants, emphasizing the significance of cyanidin in black pigments and the regulation of this coloration by R2R3-MYB transcription factors such as PeMYB7, PeMYB11, and CsMYB90, offering insights into plant color regulation and for the development of black-colored cultivars. In *Ficus virens*, Ma et al. [30] linked extended red-leaf periods to anthocyanin content and the MYB gene FvPAP1. Variations in FvPAP1 expression influence red leaf duration, providing molecular targets for breeding ornamental and woody plants with enhanced red-leaf traits.

Finally, regarding topic (v), focused investigations into plant-specific functions also contribute to agricultural advancements. Zhou et al. [31] reviewed the role of kinetochore proteins in plant meiosis, exploring their potential for genetic manipulation in plant breeding. Chen et al. [32] studied citric acid regulation in citrus, identifying citrate synthase (CS) and ATP citrate-pro-S-lyase (ACL) as key genes influencing acidity in low-acid and high-acid varieties. Sharma et al. [33] examined genetic and epigenetic stability in lingonberry during tissue culture and propagation, highlighting that while the plant retained genetic stability, it exhibited epigenetic variability under both in vitro and ex vitro conditions.

In conclusion, the papers in this Special Issue present innovative findings that advance our understanding of molecular breeding and genetics in plants. I extend my gratitude to all the authors who contributed their manuscripts, as well as to the *CIMB* editorial team, academic editors, and reviewers for their invaluable collaboration. I would also like to inform you that the 2nd edition of this Special Issue, ‘Molecular Breeding and Genetics Research in Plants, 2nd Edition’, has been launched (https://www.mdpi.com/journal/cimb/special_issues/6I87FSK9NV, accessed on 26 December 2024). We invite you to send your original articles and reviews to the new edition of this Special Issue of *CIMB*.
